# Dr. West’s Paper on Continued Fevers in Texas

**Published:** 1889-06

**Authors:** 


F. E. DANIEL, M. D., EDITOR AND PUBLISHER.

THIS JOURNAL, by unanimous vote of the members, was made the Official Organ of the Austin District Medical Society.

COLLABORATORS:

*Dr. R. M. Swearingen, Austin.*

*Prof. B. E. Hadra, M. D., Galveston.*

*Prof. Geo. Cupples, M. D., San Antonio.*

*Dr. T. C. Osborn, Cleburne, Texas.*

*Dr E. J. Doering, Chicago.*

*Dr. E. J. Beall, Fort Worth.*

*Dr Odo Betz, Germany.*

*Dr. J. W. McLaughlin, Austin.*

*Dr. T. J. Tyner, Austin.*

*Prof. J. F. Y. Paine, M. D., Galveston.*

*Dr. R. H. L. Bibb, Mexico.*

*Dr. T. J. Bennett. Austin.*

*Dr. Bat Smith, Wharton.*

*Dr. E. Meierhof, New York.*

*Prof. W. B. Rogers, M. D., Memphis, Tenn.*

### DR. WEST'S PAPER ON CONTINUED FEVERS IN TEXAS.

### AN EXPLANATION.

Dr. H. A. West, Professor of the Theory and Practice of Medicine in the Texas Medical College, at the San Antonio meeting of the State Medical Association, it will be remembered, read an excellent paper on "Continued Fevers in Texas." The paper elicited an animated discussion and a great variety of opinion was drawn out. The Secretary having his attention frequently diverted by applications for information on a variety of subjects as is usually the case the first day of the session—caused by the arrival of delegates, etc.—was unable to follow the paper and discussion as carefully as he could wish, but trusted to the stenographer of the *Express* to get a correct version of it. This gentleman must have gotten badly "rattled" by the discussion, for he reported in the *Express* that Dr. West, in his paper had taken the ground that "there is no typhoid fever in Texas"!

Nothing could have been further from Dr. West's mind. He said "that typhoid fever does prevail in Galveston, and elsewhere in Texas cannot be denied, notwithstanding there are some physicians who do not meet with it in practice"; and the Doctor even intimated that many cases of fever called by other names and treated as "catarrhal" or "malarial" or "typho-malarial" fever, were in reality, mild cases of true typhoid, not recognized as such in consequence of the absence of one or more of the well known symptoms of typhoid fever. He asserted that a description of such fever being sent to Dr. Flint was pronounced typhoid fever.

In the preparation of the minutes for the May Number of the JOURNAL, we clipped largely from the *Express*, following the short-hand reporter's record—and amongst other things, owing entirely to inadvertence on our part, we unfortunately failed to correct the statement above referred to; circumstances were such that we could not give our usual careful attention to the proof, and hence this unfortunate misrepresentation of Dr. West occurred in the May Number. We owe, and hereby tender Dr. West many apologies, and desire to correct the false impression which a reading of the minutes undoubtedly has caused.

Relying, later, upon our own notes, in preparing the minutes for the Transactions, we got Dr. West correctly reported, with regard to what he said about typhoid fever. But, the stenographer went on to say, in conclusion: "the consensus of opinion [elicted in discussion] was antagonistic to the opinions advanced by Dr. West." This, we reproduced, and thought it correct, for, the impression made on our mind was—distracted as our attention was at that particular time—that Dr. West's paper had awakened considerable opposition; that the majority of the speakers differed with him on some point or points by us not clearly understood. The impression was the more firmly fixed in our mind by the fact that after the close of the debate some member moved that "Dr. West be permitted to prepare a defense of his paper."

The doctor claims that the secretary and the stenographer are entirely wrong, and that his position was "strongly supported and his paper fully endorsed" by the members present.

Here is an unpleasant dilemma. Not for the world would we do Dr. West injustice in reporting on his paper, but we were honestly under the impression that many of the speakers differed with him. This is only as may have been expected. Dr. Paine living in the same city with him (Galveston) differed with him in toto as regards typhoid; small wonder then if other speakers living in remote parts of the State should have dissimilar experience—for Texas is a territory of 750 by 850 square miles—and presents a great diversity of climate, soil, occupations and surroundings of inhabitants, hygiene, etc. Hence it would be quite natural to suppose that many physicians differed with Dr. West, having dissimilar experience.

We would be glad if any and all the gentlemen who participated in the discussion of this paper, would drop us a line, stating their recollection of the general sentiment expressed; and especially, whether, upon the whole, Dr. West's views were endorsed, or whether, as we and the stenographer of the *Express* have it, "the consensus of opinion was antagonistic to the views advanced by the author."

If we are wrong we certainly are open to conviction, and desire to be set right—and of all things, to do Dr. West justice; if we are right, it is our duty to stand by the record, however unpleasant it may be to have to differ with our friend, the author.

It is said that during his imprisonment, Sir Walter Raleigh attempted to write a history of the world. A fight occurred in the street, in full view of his cell, and the account of it given in the papers by several witnesses differed, and did not, at all, tally with his recollection of it. On this, he gave up the work, saying, "if I cannot faithfully record what I have, myself seen, how can I record that which I have never seen."

We have written to several of the gentlemen who discussed Dr. West's paper. One reply has been received—from one of the oldest and most intelligent members, and *he* misunderstood Dr. West, even worse than the Doctor claims that we did !

